# 免疫组化法检测NSCLC患者*EGFR*突变的相关研究

**DOI:** 10.3779/j.issn.1009-3419.2015.04.01

**Published:** 2015-04-20

**Authors:** 杰 王, 畅 刘, 殿胜 钟, 东波 徐, 超 宁, 晴 马

**Affiliations:** 1 300052 天津，天津医科大学总医院肿瘤科 Department of Medical Oncology, Tianjin Medical University General Hospital, Tianjin 300052, China; 2 300070 天津，天津医科大学病理学教研室 Department of Pathology, Tianjin Medical University, Tianjin 300070, China

**Keywords:** 肺肿瘤, 表皮生长因子受体, 突变, 免疫组化法, 液相芯片法, Lung neoplasms, Epidermal growth factor receptor, Mutation, Immunohistochemistry, Liquid chip technology

## Abstract

**背景与目的:**

存在表皮生长因子受体（epidermal growth factor receptor, *EGFR*）突变的非小细胞肺癌（non-small cell lung cancer, NSCLC）患者对EGFR酪氨酸激酶抑制剂（tyrosine kinase inhibitor, TKI）治疗有良好反应。与检测*EGFR*突变的分子水平手段相比，免疫组织化学法（immunohistochemistry, IHC）价格低廉，操作简便、迅速，易开展。本研究旨在探索免疫组化法检测EGFR突变的准确性。

**方法:**

选取97例NSCLC患者的手术或组织活检标本行EGFR特异性抗体的免疫组化染色，分析染色阳性标本的临床病理特征，并继续接受液相芯片检测验证是否存在突变；新收集40例被证实为*EGFR*突变的手术标本接受免疫组化染色，计算免疫组化法检出突变灵敏度。

**结果:**

97例NSCLC标本，17例染色阳性，染色阳性标本好发于女性、腺癌、不吸烟患者中，其染色阳性标本中，76.9%实际存在突变。40例*EGFR*突变标本中，免疫组化法检出突变的灵敏度为40%。

**结论:**

免疫组化法染色评分为强阳性的标本结果准确，但该方法灵敏度不甚理想，不同研究者所得结果差异较大，临床推广是否可行仍有待进一步探讨。

## Abstract (en)

**Background and objective:**

Patients with non-small cell lung cancer (NSCLC) harboring mutations of the epidermal growth factor receptor (EGFR) respond well to EGFR-tyrosine kinase inhibitor therapy. Immunohistochemistry (IHC) is a simple and widely used technique in clinical pathology laboratories. IHC also features cost effectiveness and rapid detection of *EGFR* mutations compared with molecular methods. This study aims to determine the accuracy of IHC for *EGFR* mutation detection in NSCLC.

**Methods:**

Specimens (obtained from surgery or biopsy) from 97 NSCLC cases were stained through IHC with mutation-specific antibodies. The clinicopathological features of patients with positive immunostaining results were analyzed. Positive specimens were subjected to liquid chip technology to detect the actual EGFR status. Forty NSCLC specimens obtained from surgery and confirmed to have *EGFR* mutations through liquid chip technology were collected. These specimens were then subjected to IHC analyses with mutation-specific antibodies. The sensitivity of IHC in detecting *EGFR* mutations was calculated.

**Results:**

Seventeen of the 97 NSCLC specimens were stained positive, and positive results were mostly observed in females, patients with adenocarcinoma, and non-smokers. About 76.9% of specimens with positive IHC results harbored mutations. The sensitivity of IHC was 40% among the 40 cases identified as containing *EGFR* mutations through liquid chip technology.

**Conclusion:**

The strong positive immunostaining result is accurate, but the sensitivity of the method may not be optimal and significantly varies in different studies. The widespread application of IHC in clinics must be further investigated.

表皮生长因子受体（epidermal growth factor receptor, EGFR）的酪氨酸激酶结构域由18-24外显子编码，目前发现，超过90%的*EGFR*基因突变位于19-21外显子^[1]^。其中发生在19外显子的E746_A750缺失突变和21外显子上的L858R点突变所占比例最多，被称为经典型突变^[2, 3]^。

目前检测*EGFR*突变的方法繁多，主要是以DNA分子为基础的检测技术，可以分为两大类：普筛方法和靶向方法。普筛方法能检测所有*EGFR*突变，包括已知类型突变和未知的新型变异突变，最为经典的代表是DNA直接测序法，但因其操作繁琐、耗时长，对取材和技术要求较高，要求样本突变拷贝数含量大于30%，对环境和操作者有危害而限制了其在临床中的应用^[4]^。靶向方法仅能检测目前已知的*EGFR*突变，对未知的少见型突变无法测出，包括，特异性探针的实时定量聚合酶链式反应、扩增阻滞突变系统（amplified refractory mutation system, ARMS）技术等^[5, 6]^。这些方法因其价格昂贵，对实验环境、操作人员水平及设备要求高，影响了在临床上的广泛推行。

2009年，Yu等^[7]^用抗E746_A750del突变抗体和抗L858R点突变抗体的免疫组化法检测非小细胞肺癌（non-small cell lung cancer, NSCLC）标本*EGFR*突变，灵敏度92%，特异度99%。此后，不同研究者所得结果差异较大，灵敏度低可至24%^[8]^，高可达100%^[9]^，特异度范围介于77%-100%^[8-17]^。本文通过实验设计，旨在评价免疫组化法检测*EGFR*突变的准确性。

## 1 材料与方法

### 1.1 标本资料与方法

收集天津医科大学总医院2010年12月-2012年10月收治的NSCLC患者手术切除或组织活检标本97例，标本取材前患者未经EGFR酪氨酸激酶抑制剂（tyrosine kinase inhibitor, TKI）治疗，有基本临床资料并可随访病历。患者的平均年龄63岁（34岁-86岁）；其中，男性59例，女性38例；有吸烟史者52例，无吸烟史者45例；鳞癌30例，腺癌60例，大细胞癌7例；根据2009年国际抗癌联盟（Union for International Cancer Control, UICC）肺癌分期标准进行TNM分期，Ⅰ期23例，Ⅱ期23例，Ⅲ期21例，Ⅳ期30例。上述标本接受*EGFR*突变特异性抗体的免疫组化染色，染色阳性标本继续接受液相芯片技术检测，验证标本是否确实存在突变。

另收集天津医科大学总医院2012年2月-2013年9月经液相芯片法检测验证的40例存在*EGFR* 19或21外显子突变的手术组织蜡块。其中包含20例*EGFR* 19外显子缺失突变标本，病理资料证实均为腺癌；20例*EGFR* 21外显子点突变标本，病理资料证实17例为腺癌，3例为鳞癌，术前未经EGFR-TKIs治疗。上述标本接受*EGFR*突变特异性抗体免疫组化染色。

### 1.2 免疫组织化学染色步骤

切片常规二甲苯脱蜡，经各级浓度乙醇水化。取一定量pH 6.0柠檬酸盐缓冲液（北京中杉金桥生物技术有限公司），加入微波盒中，医用微波炉中火加热切片3 min×2次进行抗原修复，凉至室温40 min。每张切片加1滴3%H_2_O_2_，室温下孵育10 min。加1滴稀释倍数为1:100的第一抗体[抗E746_A750del突变单克隆兔抗体（6B6）和抗L858R突变单克隆兔抗体（43B2）；美国Cell Signaling Technology公司]，4 ℃冰箱过夜。而后加辣根酶标记的第二抗体（北京中杉金桥生物技术有限公司），4 ℃温箱30 min-40 min。每张切片加1滴新鲜配制的DAB液显色20 min，用淡苏木素复染细胞核30 s，各级浓度乙醇快速脱水，二甲苯透明，中性树脂封片。晾干后观察。

### 1.3 染色评判标准

以肿瘤细胞胞膜和（或）胞浆着色为基准，在低倍率对染色的强度和染色细胞百分比进行评估，随机观察10个高倍视野，根据切片中阳性细胞染色强度及百分比将表达结果分为0、1+、2+、3+四个等级。0：无染色或 < 10%肿瘤细胞染色；1+：10%-50%肿瘤细胞浅染或中度染色；2+：10%-50%肿瘤细胞强染；3+： > 50%肿瘤细胞任意强度染色。以0为阴性，1+、2+、3+为阳性。本结果由天津医科大学总医院病理科三位老师各自单独评分，结果经汇总，对有差异的评分经复核商讨后得到最终一致结果。

### 1.4 统计学方法

采用卡方检验分析2010年12月-2012年10月标本的患者年龄、性别、肿瘤分期、组织类型、吸烟情况与*EGFR*突变染色阳性标本的关系，所有统计学分析均采用SPSS 16.0进行，*P* < 0.05为差异有统计学意义。对于上述染色阳性的标本，以液相芯片法结果作为金标准，计算免疫组化法染色结果的阳性预测值（positive predictive value, PPV）。对于新收集的2012年2月-2013年9月标本，以已有的液相芯片法结果作为参考标准，计算免疫组化法检测相应突变的灵敏度（sensitivity）。

## 2 结果

### 2.1 免疫组化染色结果观察

根据所定免疫组化染色评分标准，图 1示例了100倍镜下染色对应评分。0-3+分别代表各自阴性及阳性程度。

**1:**
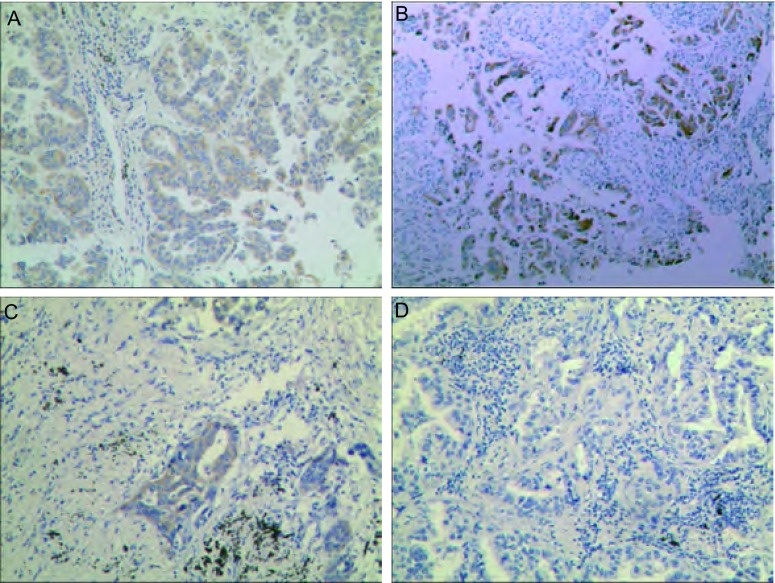
免疫组化染色结果示例图片（×100）。A：3+；B：2+；C：1+；D：0. Different degree of immunostaining results (×100). A: 3+; B: 2+; C: 1+; D: 0.

### 2.2 免疫组化染色阳性标本与肺癌临床病理生理特征关系

97例NSCLC标本中，17例免疫组化染色结果为阳性，其中抗E746_A750del抗体染色阳性标本8例（8/17, 47.06%），抗L858R抗体染色阳性标本9例（9/17, 52.94%）。免疫组化法检测*EGFR*突变率为17.5%（17/97）。

如表 1所示，不同临床特征的患者免疫组化染色阳性所占比例不同，女性阳性率高于男性（26.32% *vs* 11.86%, *P*=0.006, 44），腺癌阳性率高于其他病理类型（21.67% *vs* 10.81%, *P* < 0.001），不吸烟者阳性率高于吸烟者（28.89% *vs* 7.69%, *P*=0.001, 75），P均小于0.05，有统计学意义。但临床分期Ⅰ期-Ⅱ期与Ⅲ期-Ⅳ期阳性率（19.57% *vs* 15.69%, *P*=0.615, 84），平均年龄上下阳性率（17.78% *vs* 17.31%, *P*=0.551, 01），无统计学差异。

**1:** 免疫组化染色阳性患者的不同临床病理特征 Clinicopathologic features of the patients with positive immunostaining results

	All the patients (*n*=97)	Patients with positive IHC results	Percentage (%)	*P*
Mean age (yr)				0.551, 01
≤63	45	8	17.78	
> 63	52	9	17.31	
Gender				0.006, 44
Male	59	7	11.86	
Female	38	10	26.32	
Smoking history				0.001, 75
No	45	13	28.89	
Yes	52	4	7.69	
Clinical stage				0.615, 84
Ⅰ+Ⅱ	46	9	19.57	
Ⅲ+Ⅳ	51	8	15.69	
Histology				< 0.001
AC	60	13	21.67	
SCC	30	3	10.00	
LCC	7	1	14.29	
IHC: immunohistochemistry; AC: adenocarcinoma; SCC: squamous cell carcinoma; LCC: large cell carcinoima.				

### 2.3 染色阳性标本接受液相芯片技术检测的结果

上述17例染色阳性的标本中，由于4例组织标本无法获取，故最终获得了13例标本。如表 2所列，在这13例标本中，液相芯片法所测19外显子缺失突变者5例，21外显子点突变者5例，余3例为野生型。即在这13例免疫组化染色阳性标本中，有3例为假阳性结果（3/13, 23.1%），10例为真阳性结果（10/13, PPV=76.9%）。

**2:** 13例免疫组化染色阳性NSCLC标本的液相芯片技术检测结果 Thirteen cases of NSCLC samples with positive immunostaining results analyzed by liquid chip technology

No.	Immunostaining results		Liquid chip technology
	E746_A750del Ab	L858R Ab	
1	1+	0	wt
2	1+	0	wt
3	1+	0	wt
4	1+	0	E19del mutation
5	1+	0	E19del mutation
6	2+	0	E19del mutation
7	3+	0	E19del mutation
8	3+	0	E19del mutation
9	0	1+	E21 mutation
10	0	2+	E21 mutation
11	0	2+	E21 mutation
12	0	3+	E21 mutation
13	0	3+	E21 mutation
wt: wild type; Ab: antibody; E19del: exon 19 deletion; E21: exon 21; NSCLC: non-small cell lung cancer.			

按免疫组化染色阳性程度分（表 3）：染色为“1+”的6例，其中假阳性结果3例（3/6, 50%），实际突变3例（3/6, PPV=50%）；染色为“2+”的3例，无假阳性结果，实际3例均突变（3/3, PPV=100%）；染色为“3+”的4例，无假阳性结果，实际4例均突变（4/4, PPV=100%）。

**3:** 免疫组化法所测*EGFR*突变的阳性预测值 PPV of *EGFR* mutation by IHC

Immunostaining scores	*n*	Cofirmed mutated samples	PPV
1+	6	3	50%
2+	3	3	100%
3+	4	4	100%
Total	13	10	76.9%
EGFR: epidermal growth factor receptor; PPV: positive predictive value.			

### 2.4 40例*EGFR*突变阳性标本的免疫组化染色结果分析

在40例液相芯片法所测*EGFR* 19或21外显子突变阳性标本中，两种特异性抗体的免疫组化法染色结果为阳性的占16例，灵敏度为40%（16/40）。

在20例液相芯片法所测*EGFR* 19外显子缺失突变标本中，应用抗L858R抗体检测标本染色结果均为阴性；应用抗E746_A750del抗体检测标本染色结果为阳性的占9例，即该抗体的免疫组化法检测*EGFR* E19缺失突变的灵敏度为45%（9/20）。

在20例液相芯片法所测*EGFR* 21外显子突变标本中，应用抗E746_A750del抗体检测标本染色结果均为阴性；应用抗L858R抗体检测标本染色结果为阳性的占7例，即该抗体的免疫组化法检测*EGFR* E21突变的灵敏度为35%（7/20）。

## 3 讨论

本文首先对97例NSCLC患者的手术或组织活检标本行特异性抗体的免疫组化染色，结果示17例染色阳性，即免疫组化法检测*EGFR*突变率为17.5%，与2011年Li等^[18]^对该院208例NSCLC患者行DNA测序所得的24.5%的*EGFR*突变率大体一致。*EGFR*突变比例在不同人种中差别较大，欧美地区NSCLC患者*EGFR*突变发生率约为10%-16%，亚裔NSCLC患者的*EGFR*突变发生率约为30%-50%^[19, 20]^。在中国和日本不同研究者所行检测中，*EGFR*突变阳性比例范围在17.3%-58%之间^[18, 21-25]^。另外，*EGFR*免疫组化染色阳性的病例易发生在女性、腺癌、不吸烟患者中（*P* < 0.05），与肺癌患者年龄、临床分期等无明显关系（*P* > 0.05），上述结论均与传统共识相符^[26-28]^。进一步对上述免疫组化法染色阳性的NSCLC患者标本行液相芯片检测技术，并以后者作为评判标准，验证免疫组化法检测*EGFR*突变的准确性。17例染色阳性的标本，除去缺失的4例，剩余13例中，液相芯片法检测出10例*EGFR*突变（10/13, 76.9%），染色评分为“2+”和“3+”的标本，实际验证均存在突变，PPV均高达100%。由此推测，免疫组化法染色评分为强阳性的标本结果可靠。

由于上述13例均为免疫组化染色阳性的标本，仅可算出诊断试验的PPV，初步评价了免疫组化法预测阳性标本的准确性，因此，我们又新收集了经液相芯片法证实为突变阳性的标本，行免疫组化染色，进一步评价免疫组化法检出突变的灵敏度。结果示应用抗E746_A750del抗体检出19外显子缺失突变的灵敏度为45%（9/20）。应用抗L858R抗体检出21外显子突变的灵敏度为35%（7/20）。E746_A750del抗体检测19外显子缺失突变的灵敏度高于应用L858R抗体检测21外显子突变的灵敏度，这与Brevet^[12]^、Kato^[13]^、Kitamura^[15]^、Hofman等^[8]^研究者的实验结果相符，但与Kawahara^[29]^、Ambrosini-Spaltro^[9]^等的结果有差异，可见此结果目前尚难定论。

表 4列举了近些年来不同研究者行特异性抗体的IHC检测*EGFR* 19和21外显子突变灵敏度的情况，对总体的检测，从29.8%-80.4%波动范围较大，推测影响结果的因素较多。标本类型包括组织切片和组织芯片。Ambrosini-Spaltro等^[9]^认为，应用组织切片可能较组织芯片更完整显示出肿瘤细胞免疫组化反应情况，由此推测在其他因素相同的情况下，应用组织切片标本所得灵敏度结果较组织芯片更为理想。但Hofman等^[8]^同样应用组织切片标本进行实验，所得灵敏度结果仍偏低，本实验结果介于各研究结果之间，与免疫组化法检测*EGFR*突变实际情况大体相符。

**4:** 不同研究者免疫组化法检测*EGFR*突变灵敏度的总结 Sensitivity of IHC in detecting *EGFR* mutations from different studies

Studies	*n*	IHC methodology	Gold standard	IHC scoring criteria	Sensitivity in detecting *EGFR* mutations by IHC		
					Specific Abs in detecting E19/E21 mutations	E746-A750del Ab in detecting E19 mutations	L858R Ab in detecting E21 mutations
Kawahara^[29]^	60	Slides	DNA sequencing	4 grades, visual scoring	32/44 (72.7%)	13/21 (61.9%)	19/23 (82.6%)
Kato^[13]^	70	TMA	DNA sequencing	H score, cut off values at 20	18/30 (60.0%)	9/18 (50.0%)	9/12 (75.0%)
Kitamura^[15]^	238	TMA	DNA sequencing	4 grades, digital scoring	28/78 (35.9%)	16/41 (39.0%)	12/37 (32.4%)
Simonetti^[14]^	78	Slides	DNA sequencing	4 grades, visual scoring	45/56 (80.4%)	20/29 (69.0%)	25/27 (92.6%)
Ilie ^[30]^	61	TMA	DNA sequencing	4 grades, visual scoring	No mutations in exon 21	9/10 (90.0%)	No mutations in exon 21
Ambrosini-Spaltro^[9]^	33	Slides	DNA sequencing	4 grades, visual scoring	11/18 (61.1%)	6/12 (50.0%)	5/6 (83.3%)
Hofman^[8]^	154	Slides	DNA sequencing	4 grades, visual scoring	17/57 (29.8%)	13/36 (36.1%)	4/21 (19.0%)
Jiang^[11]^	399	Slides	TaqMan PCR	4 grades, visual scoring	118/162 (72.8%)	69/90 (76.7%)	53/76 (69.7%)
Our study	40	Slides	Liquid chip technology	4 grades, visual scoring	16/40 (40.0%)	9/20 (45.0%)	7/20 (35.0%)
TMA: tissue microarray.							

对于NSCLC患者*EGFR*突变的检测，相比分子水平的检测手段，免疫组化法因其价格低廉、操作简便、易于临床开展而受到了人们的关注。免疫组化法染色评分为强阳性的标本结果准确，但灵敏度不甚理想，临床推广是否可行仍有待进一步探讨。
